# 5-Aminolevulinic Acid-Based Sonodynamic Therapy Induces the Apoptosis of Osteosarcoma in Mice

**DOI:** 10.1371/journal.pone.0132074

**Published:** 2015-07-10

**Authors:** Yongning Li, Qi Zhou, Zheng Hu, Bin Yang, Qingsong Li, Jianhua Wang, Jinhua Zheng, Wenwu Cao

**Affiliations:** 1 School of Life Science and Technology, Harbin Institute of Technology, Harbin, 150080, China; 2 Laboratory of Photo- and Sono-theranostic Technologies and Condensed Matter Science and Technology Institute, Harbin Institute of Technology, Harbin, 150080, China; 3 Cardiovascular Institute, the First Affiliated Hospital of Dalian Medical University, Dalian, 116011, China; 4 Department of Anatomy, College of Basic Medical Sciences, Harbin Medical University, Harbin, 150080, China; 5 Materials Research Institute, the Pennsylvania State University, University Park, PA, 16802, United States of America; University of Pecs Medical School, HUNGARY

## Abstract

**Objective:**

Sonodynamic therapy (SDT) is promising for treatment of cancer, but its effect on osteosarcoma is unclear. This study examined the effect of 5-Aminolevulinic Acid (5-ALA)-based SDT on the growth of implanted osteosarcoma and their potential mechanisms *in vivo* and *in vitro*.

**Methods:**

The dose and metabolism of 5-ALA and ultrasound periods were optimized in a mouse model of induced osteosarcoma and in UMR-106 cells. The effects of ALA-SDT on the proliferation and apoptosis of UMR-106 cells and the growth of implanted osteosarcoma were examined. The levels of mitochondrial membrane potential (ΔψM), ROS production, BcL-2, Bax, p53 and caspase 3 expression in UMR-106 cells were determined.

**Results:**

Treatment with 5-ALA for eight hours was optimal for ALA-SDT in the mouse tumor model and treatment with 2 mM 5-ALA for 6 hours and ultrasound (1.0 MHz 2.0 W/cm^2^) for 7 min were optimal for UMR-106 cells. SDT, but not 5-ALA, alone inhibited the growth of implanted osteosarcoma in mice (P<0.01) and reduced the viability of UMR-106 cells (p<0.05). ALA-SDT further reduced the tumor volumes and viability of UMR-106 cells (p<0.01 for both). Pre-treatment with 5-ALA significantly enhanced the SDT-mediated apoptosis (p<0.01) and morphological changes. Furthermore, ALA-SDT significantly reduced the levels of ΔψM, but increased levels of ROS in UMR-106 cells (p<0.05 or p<0.01 vs. the Control or the Ultrasound). Moreover, ALA-SDT inhibited the proliferation of osteosarcoma cells and BcL-2 expression, but increased levels of Bax, p53 and caspase 3 expression in the implanted osteosarcoma tissues (p<0.05 or p<0.01 vs. the Control or the Ultrasound).

**Conclusions:**

The ALA-SDT significantly inhibited osteosarcoma growth in vivo and reduced UMR-106 cell survival by inducing osteosarcoma cell apoptosis through the ROS-related mitochondrial pathway.

## Introduction

Osteosarcoma is a malignant tumor threatening young adults worldwide and accounts for 20% of primary bone cancers. Currently, there are several therapeutic strategies available for treatment of osteosarcoma. However, the efficacy of these therapeutic strategies is limited and some therapeutic procedures can cause severe complications and adverse effects. Given that there is no effective therapy for treatment of osteosarcoma[1–4] new approaches to discovery of effective therapies for osteosarcoma are of great significance.

Sonodynamic therapy (SDT) was developed by Umemura et al. in 1989 for cancer treatment, and is a non-thermal method utilizing low-intensity ultrasound and sonosensitizers[5]. Sonosensitizers can selectively accumulate in tumor cells, activated by ultrasound in the targeted area and generate reactive oxygen species (ROS) to kill tumor cells [6–9]. 5-aminolevulinic acid (5-ALA) can metabolize into the biological precursor of protoporphyrin IX (PpIX) in the haeme biosynthesis pathway and 5-ALA has low toxicity and a short dark period in the cells, as compared with other sonosensitizers[10,11]. The PpIX derived from 5-ALA mainly accumulates in the mitochondria of cells[12], where the generated ROS following ultrasound can through the mitochondrial apoptotic pathway trigger vertebrate cell apoptosis[13,14].

The effects of 5-ALA-mediated sonodynamic therapy (ALA-SDT) for tumor cells have been extensively investigated[15,16]. Some studies have elucidated that ALA-SDT can induce the mitochondrial apoptotic pathway of tumor cells[15,17]. However, the molecular mechanisms underlying the action of ALA-SDT have not been clarified. In this study, we employed a mouse model of osteosarcoma and in vitro tumor cells to examine the effect of ALA-SDT on osteosarcoma cell survival and apoptosis, and to investigate the potential mechanisms by which ALA-SDT treatment induced rat osteosarcoma UMR-106 cell apoptosis.

## Materials and Methods

### Cell culture

Rat osteosarcoma UMR-106 cells were obtained from Chinese Academy of Sciences, Shanghai Institute Cell Resource Center, China. UMR-106 cells (1×10^5^~1.5×10^5^ cells/ml) were cultured in DMEM (HyClone, Logan, UT, USA) medium containing 10% fetal bovine serum (FBS), 100 units/mL penicillin, and 100 μg/mL streptomycin at 37°C in a humidified atmosphere of 5% CO_2_.

### Sonication device

**Fig 1:**
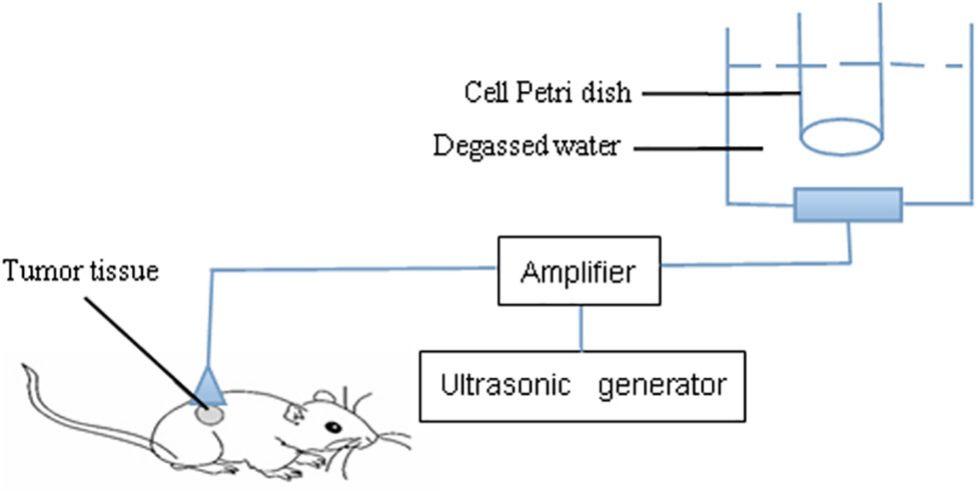
Schematic diagram of the sonication device.

The ultrasonic generator and power amplifier used in this study were designed and assembled by Harbin Institute of Technology (Harbin, China, Fig 1). The home-made ultrasonic transducer (diameter: 3.5 cm: resonance frequency: 1.0 MHz; duty factor: 10%; repetition frequency: 100 Hz) was placed in a water bath 30 cm below the cells cultured to guarantee field uniformity. The ultrasound intensity used was 2.0 W/cm^2^ *in vivo* and 3.0 W/cm^2^ *in vitro*, measured by a hydrophone (Onda Corp, Sunnyvale, CA, USA).

### Animal tumor model and treatments

Male BALB/c nude mice at 4 weeks of age were from Shanghai Laboratory Animal Center (SLAC, Shanghai, China) and housed in a specific pathogen-free facility. Individual mice were inoculated subcutaneously with 2x10^4^ UMR-106 cells in 100 μl serum-free medium near the right hip to induce solid tumors. When the tumor reached a diameter of 0.5 to 0.7 cm (about 7 days later), the tumor-bearing mice were randomly treated intravenously with 250 mg/kg 5-ALA (Sigma, St. Louis, Mo, USA) and protected from light exposure. The intracellular contents of 5-ALA-PpIX in the tumor or surrounding tissues of mice were examined longitudinally using the Leica LT-9MACIMSYSPULS (Media Cybernetics, Inc, Bethesda, MD, USA) under the activation of a 405-nm blue light source because the contents of intracellular 5-ALA-PpIX are correlated positively with the intensity of red fluorescence.

The tumor-bearing mice at seven days post inoculation were randomized into four groups (n = 10 per group): the control (Control), 5-ALA (ALA), ultrasound (Ultrasound) and SDT (SDT). The Control mice received intravenously vehicle alone and the ALA mice received 250 mg/kg 5-ALA daily. The Ultrasound group of mice were treated with ultrasound of 2.5 W/cm^2^ for eight minutes while the SDT group of mice were treated with the same dose of 5-ALA, followed by ultrasound treatment eight hours later. All mice were treated with daily for consecutive 10 days and protected from light exposure until the end of experiment. The tumor volumes in individual mice were measured using a caliper and calculated using the formula of [(π/6)a×b^2^], where a was the long diameter while b for the short diameter. All mice were sacrificed at 10 days post 5-ALA administration. The experimental protocols were approved by the Animal Care and Research Committee of Harbin Institute of Technology.

### The 5-ALA cytotoxicity and intracellular ALA-PpIX accumulation

UMR-106 cells (3×10^4^~5×10^4^ cells/ml) were cultured in 96-well plates for 6 hours and treated in sextuplicate with different doses (0.5–10 mM) of 5-ALA for varying periods (2–12 hours). During the last two-hour culture, the cells were exposed to 20 μl MTT and the absorbance of individual wells was measured at 450 nm. In addition, UMR-106 cells (3×10^4^~5×10^4^ cells/ml) were treated in sextuplicate with 2 mM 5-ALA for 2–12 hours and the contents of intracellular 5-ALA-PpIX were measured longitudinally using a fluorescence spectrophotometer (USB2000; Ocean Optics Inc, Dunedin, FL, USA) and fluorescence microscope (Olympus, Tokyo, Japan).

### Ultrasound treatment *in vitro*

UMR-106 cells (3×10^4^~5×10^4^ cells/ml) were cultured in 35 mm dishes overnight and randomly treated in sextuplicate with vehicle alone as the Control (C), with 5-ALA as the A group, with ultrasound as the Us group or with SDT as the SDT group. The control cells were treated in sextuplicate with vehicle, the A group with 2 mM 5-ALA, the Us group with ultrasound of 2.0 W/cm^2^ for 7 min at 30 cm from the transducer and the SDT group with both the same dose of 5-ALA and ultrasound for 6 hours. The viability of individual groups of cells was determined by MTT.

### Transmission electron microscopy (TEM) analysis

Individual group of cells were harvested and centrifuged, after being washed with PBS (0.01M pH7.2~7.4), the cells were embedded with 2% agar in 1% glucose medium, fixed with 2.5% glutaraldehyde for 2 hours and then further fixed with osmium tetroxide. Subsequently, the blocks were dehydrated in gradient alcohol, and embedded in Epon812. The cells were then cut into ultra-thin sections (8 nm), and stained with uranium acetate. The sections were observed and photoimaged under a TEM (Hitachi, Tokyo, Japan).

### TUNEL assay of apoptotic cells *in vivo*

The cell apoptosis in the tumors was assessed by the terminal deoxyribonucleotide transferase mediated nick-end labelling (TUNEL) assay using an *in situ* apoptotic detection kit (Roche, Switzerland), according to the manufacturer’s instructions. Briefly, the tumor sections (5 μm) from individual mice were subjected to TUNEL assay and stained with diaminobenzene (DAB) for 10 min. The sections were examined under a light microscope (Nikon, Tokyo, Japan) and the cells with positively tan or brown stained particles in their nuclei were apoptotic cells. The apoptotic index was calculated as the numbers of TUNEL-positive cells divided by the total number of cells in 10 randomly selected high-power fields (HPF, magnification × 400), and at least 1000 tumor cells were counted for individual mice.

### Immunohistochemistry

Tumor tissues were fixed in 4% PFA, dehydrated with graded ethanol and paraffin-embedded. The tumor tissue sections (4 μm) were deparaffined, rehydrated and subjected to antigen-retrieval by heating the sections in citrate buffer (0.01 M, pH 6.0) for 20 minutes, followed by treating them with 3% H_2_O_2_ to block endogenous peroxidase at room temperature. After being blocked with 10% goat serum, the sections were stained with rabbit polyclonal anti-Bcl-2 (1:200), anti-Bax (1:200), anti-P53, and mouse monoclonal anti-caspase-3 (1:200) (1:200; Santa Cruz Biotechnology, Santa Cruz, CA, USA) at 4°C overnight. The sections were washed with PBS for 3 times, and the bound antibodies were detected with goat anti-rabbit or goat anti-mouse secondary antibodies and visualized with DAB, followed by counterstaining with hematoxylin. Finally, the sections were examined under a light microscope and immunopositively stained cells was quantified with integrated optical density (IOD) values using Image Pro Plus (IPP) software 6.0 (Media Cybernetics, Bethesda, MD, USA).

Additional immunohistochemistry was performed to detect proliferative cells in tumor sections using mouse monoclonal anti-proliferating cell nuclear antigen (PCNA) antibody (sc-25280; 1:200; Santa Cruz Biotechnology). A total of 10 HPF (magnification × 400) were selected randomly to evaluate the numbers of positive anti-PCNA stained cells and the proliferation index was calculated as the numbers of PCNA positive cells divided by the total number of cells in HPF selected.

### Flow cytometry

UMR-106 cells (5×10^4^ cells /well) were cultured in 35 mm dishes overnight and treated in octuplicate with vehicle alone, with 2 mM 5-ALA, ultrasound at 2.0 W/cm^2^ (1.0 MHz) for seven minutes, or with both the same doses of 5-ALA and ultrasound. The cells were cultured for six hours and stained with 5 μL Annexin-V-FITC for 15 minutes and 10 μL propidium iodide (PI, Key Gen Biotech, Beijing, China) for 5 minutes. After being washed, the cells of each sample were analyzed by flow cytometry. The percentages of apoptotic and necrotic cells were analyzed using CELL Quest software (BD Biosciences, San Jose, USA).

### Fluorescent staining and fluorospectrophotometer assays

The impact of treatment with 5-ALA and/or ultrasound on the mitochondrial membrane potential (ΔψM) was assessed by fluorescent staining and fluorospectrophotometer assays using fluorescent probe jc-1 (Invitrogen). UMR-106 cells (5×10^4^ cells/well) were cultured in 35 mm dishes overnight and treated in octuplicate with vehicle alone, with 2 mM 5-ALA, ultrasound at 2.0 W/cm^2^ (1.0 MHz) for 7 minutes, or with both the same doses of 5-ALA and ultrasound. The cells were cultured for six hours and stained with 10 mg/ml jc-1 for 20 minutes at 37°C in the dark. After being washed, the cells were examined under a fluorescent microscope. The red–orange fluorescence reflected a potential-dependent aggregation in the mitochondria and the green fluorescence, the monomeric form of jc-1, in the cytosol indicated the mitochondrial membrane depolarization. The fluorescence intensity was measured using a fluorospectrophotometer (Olympus Corporation, Tokyo, Japan) at 488 nm excitation and 530 nm (green) and 590 nm (red) emission wavelengths.

In addition, the impact of treatment with 5-ALA and/or ultrasound on the production of ROS in individual groups of cells was determined by fluorescent staining and fluorospectrophotometer assays using 2′-7′-dichlorofluorescin diacetate (DCFH-DA). Briefly, the different groups of cells were treated as described above for 7 minutes and stained with 10 μM DCFH-DA at 37°C for 30 min. After being washed, the intensity of fluorescent signals was detected using a fluorescence spectrophotometer at emission from 488 to 515 nm. Furthermore, the cells were examined under a fluorescent microscope.

### Statistical analysis

Statistical analysis was performed using SPSS 13.0 software. All data were expressed as the means ± standard deviation (SD). The difference among the groups were analyzed with one way ANOVA and post hoc with Fisher's least significant difference (LSD). A p value of <0.05 was considered statistically significant.

## Results

### Pre-treatment with 5-ALA enhances the effect of SDT on inhibiting the growth of implanted osteosarcoma in mice

**Fig 2:**
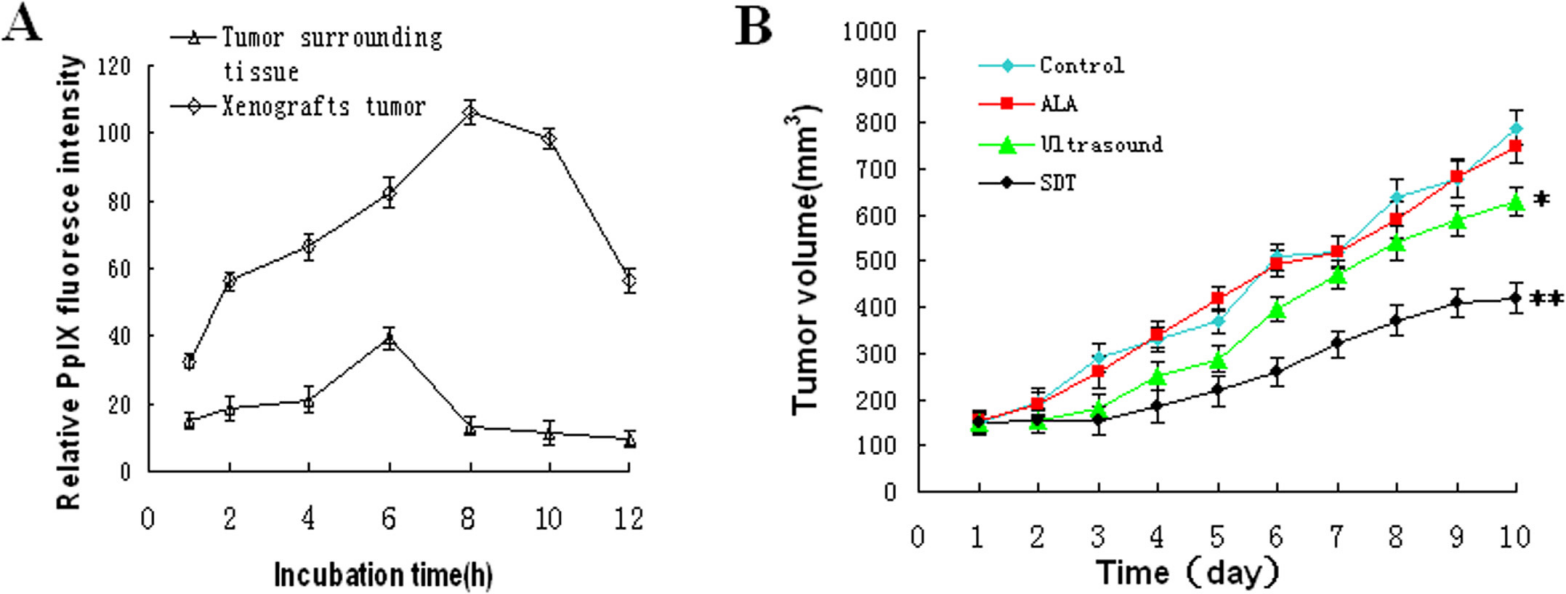
The dynamics of 5-ALA metabolism in tumors and ALA-SDT mediated inhibition on the growth implanted osteosarcoma in mice. BALB/c nude mice were inoculated with UMR-106 cells to induce solid tumor and the tumor-bearing mice were treated with 250 mg/kg 5-ALA. The contents of generated PpIX in the tumors or surrounding tissues of individual mice were evaluated longitudinally at the indicated time points. In addition, the tumor-bearing mice were treated with vehicle alone as the Control, with 5-ALA (ALA), ultrasound or both as the ALA-SDT daily for 10 consecutive days. The volumes of tumor were monitored daily. Data are expressed as the mean ± SD of individual groups (n = 3 per group for the measurement of PpIX, n = 10 per group for measurement of in vivo tumor growth). A. The dynamics of PpIX generation in the tumor. B. The growth of implanted tumors in mice. *P<0.05, **p<0.01 vs. the Control.

To determine the effect of ALA-SDT, we first determined the metabolic dynamics of 5-ALA in vivo. BALB/c nude mice were inoculated with UMR-106 cells to induce solid tumors. The tumor-bearing mice were treated with 5-ALA and the contents of generated PpIX in the tumors and surrounding regions of individual mice were measured using the Leica LT-9MACIMSYSPULS. As shown in Fig 2A, the red fluorescence in the skin of surrounding regions of individual mice peaked at 6 hours post injection and declined. In contrast, the red fluorescent signals in the xenograft tumors gradually increased and peaked at 8 hours post injection and declined. As a result, the ratios of signals in the tumor to that in the surrounding regions that reflected the special signals were the greatest at 8 hours post injection, suggesting the best time for SDT.

Next, we tested the effect of ALA-SDT on the growth of implanted tumors in vivo. The tumor-bearing mice were randomly treated with vehicle as the Control, with ALA, ultrasound alone or both 5-ALA and ultrasound as the SDT group and the growth of implanted tumors were monitored in Fig 2B. The tumor volumes in the ALA-treated mice were similar to that of the Controls and the tumor volumes in the Ultrasound group were significantly smaller than that of the Controls, indicating that treatment with ultrasound alone, but not ALA, inhibited the growth of implanted osteosarcoma *in vivo*. More importantly, the tumor volumes in the mice received ALA and ultrasound were significantly smaller than that of the Controls (p<0.01). Hence, pre-treatment with 5-ALA enhanced the antitumor effect of ultrasound.

### Optimization of experimental conditions for ALA-SDT mediated cytotoxicity against UMR-106 cells

**Fig 3:**
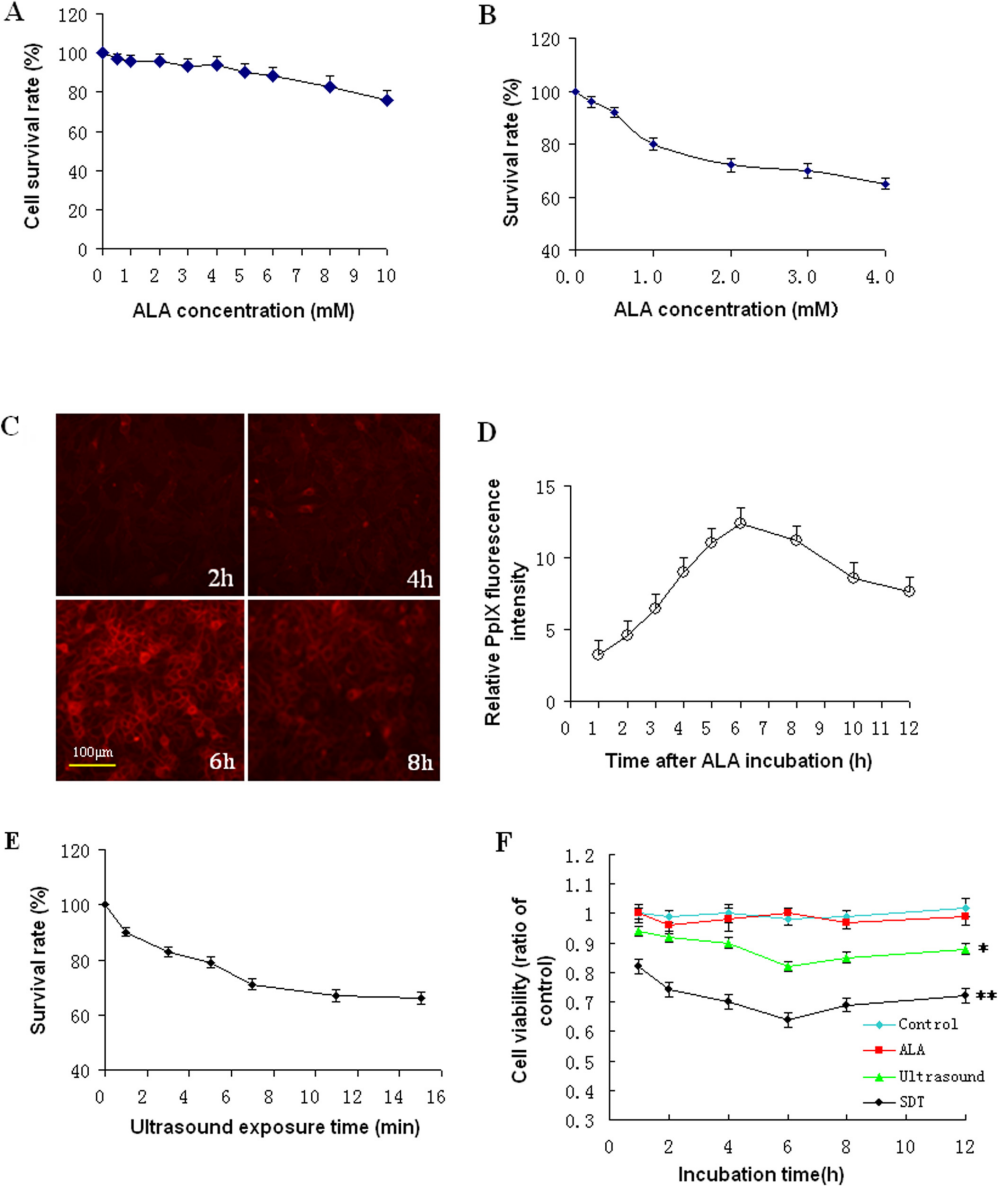
Optimization of ALA-SDT for UMR-106 cells in vitro. UMR-106 cells were treated with 0–10 mM 5-ALA for 2–12 house and during the last 2-h culture, the cells were exposed to MTT for determine the optimal concentration of 5-ALA by the survival of cells. Furthermore, the cells were treated with 0–4 mM and exposed to ultrasound at 2.0 W/cm^2^ for 5 minutes, followed by determining the survival of cells. In addition, the cells were treated with 2 mM 5-ALA for 2–12 hours and the intracellular contents of PpIX in individual groups of cells were determined longitudinally using a fluorescent microscope and spectrophotometer. Moreover, the cells were treated with 2 mM 5-ALA and then with ultrasound for 1–15 minutes, followed by determining the survival rates of cells. Finally, the cells were treated with vehicle as the Control, 2 mM 5-ALA or/and ultrasound for 7 minutes and the viability of individual groups of cells was determined by MTT. Data are representative fluorescent images or expressed as the mean ± SD of individual groups of cells from three separate experiments. A. The dose effects of 5-ALA on the survival of cells. B. The dose effect of ALA_SDT on the survival of cells. C. The fluorescent images (magnification x 400) of intracellular PpIX. D. The quantitative analysis of intracellular PpIX. E. The time effect of ALA-SDT on the survival of cells. F. The effect of ALA-SDT on the viability of cells. *P<0.05, **p<0.01 vs. the Control.

To test the effect of ALA-SDT, we began to optimize the concentrations of 5-ALA and found that treatment with 1–4 mM 5-ALA did not modulate the cell viability in vitro and treatment with increased concentrations of 5-ALA slightly reduced the cell viability (Fig 3A). Furthermore, we tested the effect of 0–4 mM 5-ALA and ultrasound on the viability of UMR-106 cells and we found that treatment with 2–4 mM 5-ALA and ultrasound dramatically reduced the viability of UMR-106 cells (Fig 3B). Hence, we selected 2 mM 5-ALA for further experiments. In addition, we determined the dynamic metabolism of 5-ALA in UMR-106 cells and found that the contents of generated PpIX peaked at 6 hours post treatment in UMR-106 cells (Fig 3C and 3D). Moreover, we optimized the ultrasound time period and we found that treatment with 2 mM 5-ALA and ultrasound for 7 minutes reached a great reduction in the viability of UMR-106 cells (Fig 3E). Collectively, our data indicated that treatment with 2 mM 5-ALA for 6 hours and ultrasound for 7 minutes were optimal for testing the effect of ALA-SDT on the survival of UMR-106 cells in vitro. Actually, we detected that the survival rates of cells that had been treated with 2 mM 5-ALA were similar to that of the Controls and the survival rates of cells treated with ultrasound alone were significantly lower than that of the Controls beginning (p<0.05, Fig 3F). Interestingly, the survival rates of cells in the SDT group were further reduced to 64.4%±3.1%, as compared with that of the Controls (p<0.01). Together, our data indicated that treatment with 5-ALA enhanced the cytotoxicity of ultrasound against UMR-106 cells *in vitro*.

### Treatment with 5-ALA enhances ultrasound-mediated UMR-106 cell apoptosis in vitro

**Fig 4:**
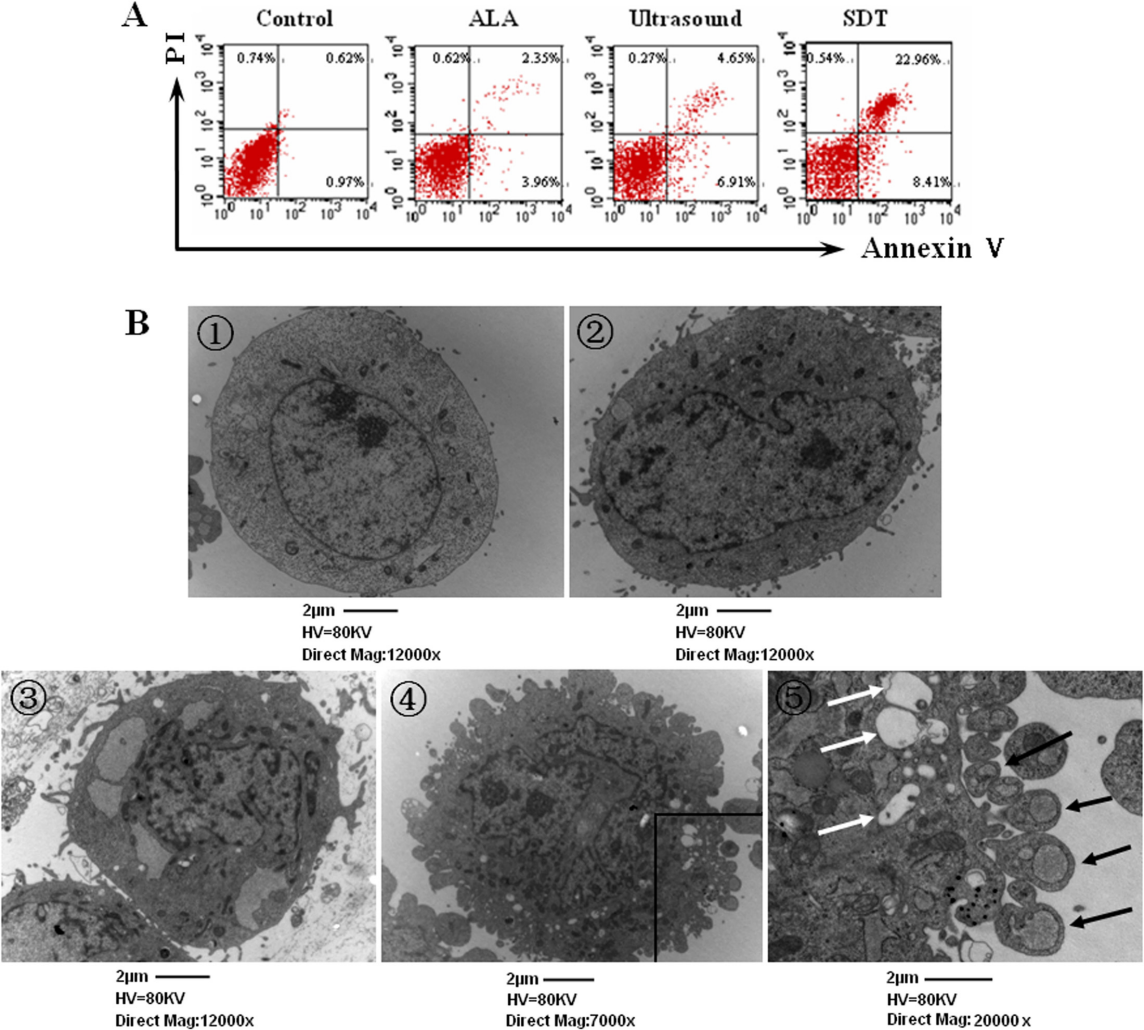
ALA-SDT induces osteosarcoma cell apotosis in vitro. UMR-106 cells were treated with vehicle (Control), 2 mM 5-ALA or/and ultrasound for 7 minutes, cultured for 6 hours and the percentages of apoptotic cells were determined by flow cytometry. In addition, the cells damages of individual groups of cells were characterized by TEM. Data are representative images of individual groups of cells from three separate experiments. A. Flow cytometry analysis of apoptotic cells. B. TEM analysis of cell damages (Magnification×20000). The white arrows indicate apoptotic bodies.

To understand the therapeutic mechanisms underlying the action of ALA-SDT, we determined the effect of treatment with ALA and/or ultrasound on the frequency of apoptotic UMR-106 cells in vitro. We found that treatment with 5-ALA alone did not significant increase the frequency of apoptotic UMR-106 cells and treatment with ultrasound alone did increase the frequency of apoptotic UMR-106 cells (11.56% vs. 1.59%, p<0.05, Fig 4A). Treatment with both 5-ALA and ultrasound further significantly increased the frequency of apoptotic UMR-106 cells (31.37%, p<0.01 vs. the Ultrasound or Controls). Hence, the ALA-SDT triggered the apoptosis of UMR-106 cells, contributing to their inhibition of tumor growth in vivo and cytotoxicity in vitro.

TEM analysis revealed that there was no obvious cell damage and apoptotic body in the Control and ALA groups of cells (Fig 4B). In contrast, the cells in the ultrasound group displayed varying sizes and irregular shapes, accompanied by decreased superficial microvillus and swollen mitochondria and endoplasmic reticulum, indicating cell damages. Furthermore, the SDT group of cells exhibited more severe damages with many apoptotic bodies and different sizes of vacuoles and organelles in the cytoplasm as well as disrupted cell membranes and nuclei. These data provided a separate line of evidence to demonstrate that the ALA-SDT enhanced UMR-106 cell apoptosis in vitro.

### Treatment with 5-ALA enhances ultrasound-modulated ΔψM and ROS production in UMR-106 cells

**Fig 5:**
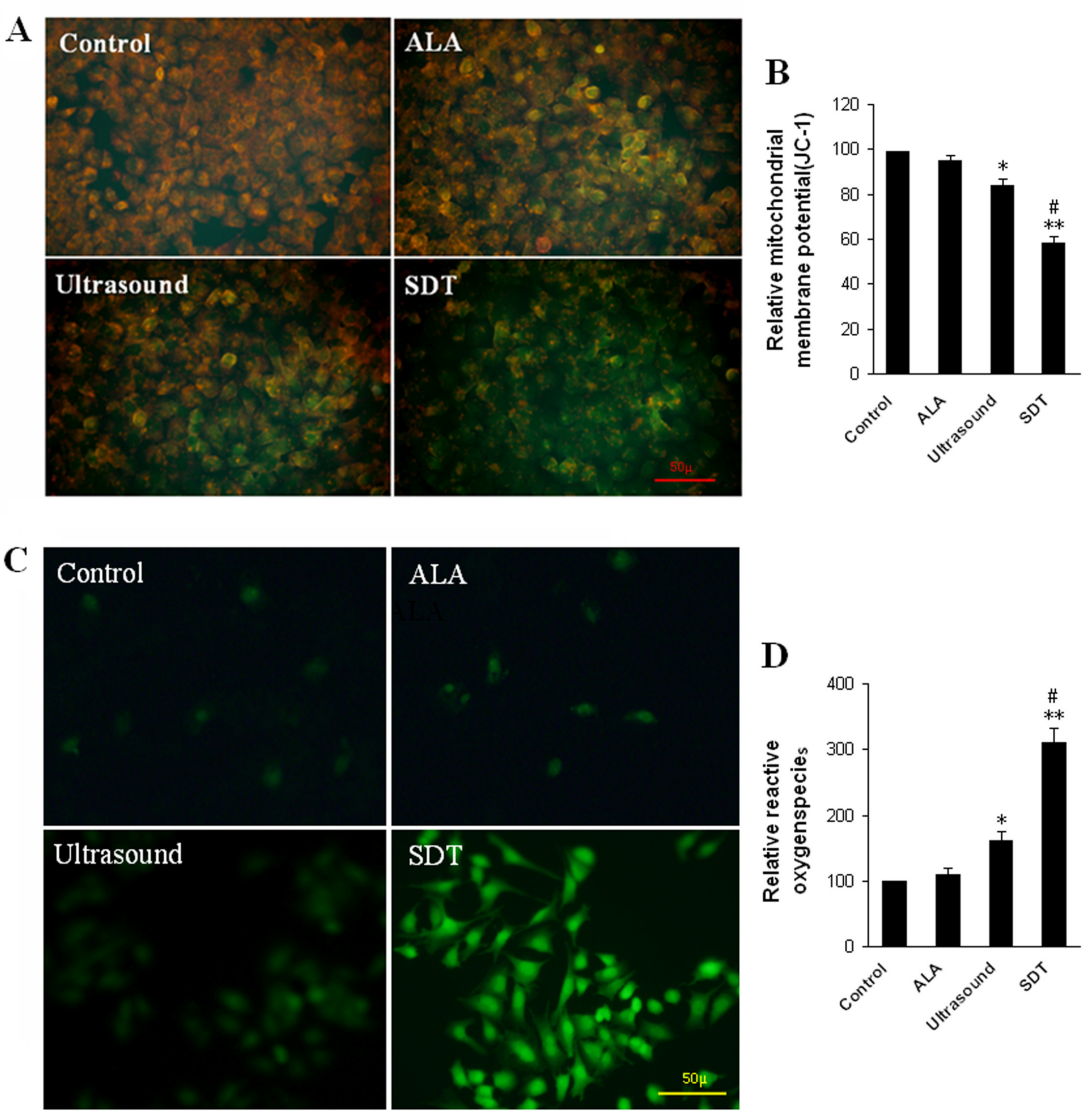
ALA-SDT significantly reduces the mitochondrial membrane potential (ΔψM) and promotes high levels of ROS production in UMR-106 cells. UMR-106 cells were treated, as described above, and stained with JC-1 or DCFH-DA. Subsequently, the changes in the ΔψM and ROS production in individual groups of cells were determined by fluorescent imaging and spectrophotometer. Data are representative images (magnification x400) or expressed as the means ± SD of individual groups of cells from three separate experiments. A. Fluorescent images of JC-1 staining. B. Quantitative analysis of ΔψM. C. Fluorescent images of ROS production. D. Quantitative analysis of ROS production. *P<0.05, **p<0.01 vs. the Control. #p<0.05 vs. the ultrasound alone.

Treatment with ultrasound can reduce the ΔψM and induce ROS production[15,18]. To further understand the therapeutic mechanisms of ALA-SDT, UMR-106 cells were treated with vehicle, 5-ALA, ultrasound alone or both 5-ALA and ultrasound, and labeled with jc-1 dye. The intensity of green and red-orange fluorescence of jc-1 were examined under a fluorescent microscope (Fig 5A) and measured by a fluorospectrophotometer (Fig 5B). The levels of ΔψM in the ultrasound group were significantly lower than that of the Control or ALA group (p<0.05) and the levels of ΔψM in the cells treated with both 5-ALA and ultrasound were further reduced to 40.2 ± 2.6%, which were significantly lower than that of the Control (p<0.01) and ultrasound (p<0.05). Hence, the ALA-SDT significantly reduced the ΔψM in UMR-106 cells.

Analysis of ROS production showed significantly increased numbers of cells with strong intensity of DCFH-DA staining in the ALA-SDT group and relative less numbers of DCFH-DA positive cells with relative low intensity of fluorescent signals in the ultrasound group (Fig 5C). However, there were a few positive DCFH-DA staining cells in the Control or ALA group. Quantitative analysis revealed similar levels of ROS production in both the Control and ALA groups (100 and 112.8 ± 9.6%, Fig 5D). The levels of ROS in the ultrasound group (168.3 ± 14.5%) were significantly higher than that of the Control (p<0.05), but lower than that in the ALA-SDT group (312.4± 20.4%, p<0.05). Therefore, these data indicated that treatment with ALA enhanced ultrasound-reduced ΔψM, and ultrasound-induced ROS production in UMR-106 cells in vitro.

### The ALA-SDT inhibits UMR-106 cell proliferation and induces UMR-106 cell apoptosis in vivo

**Fig 6:**
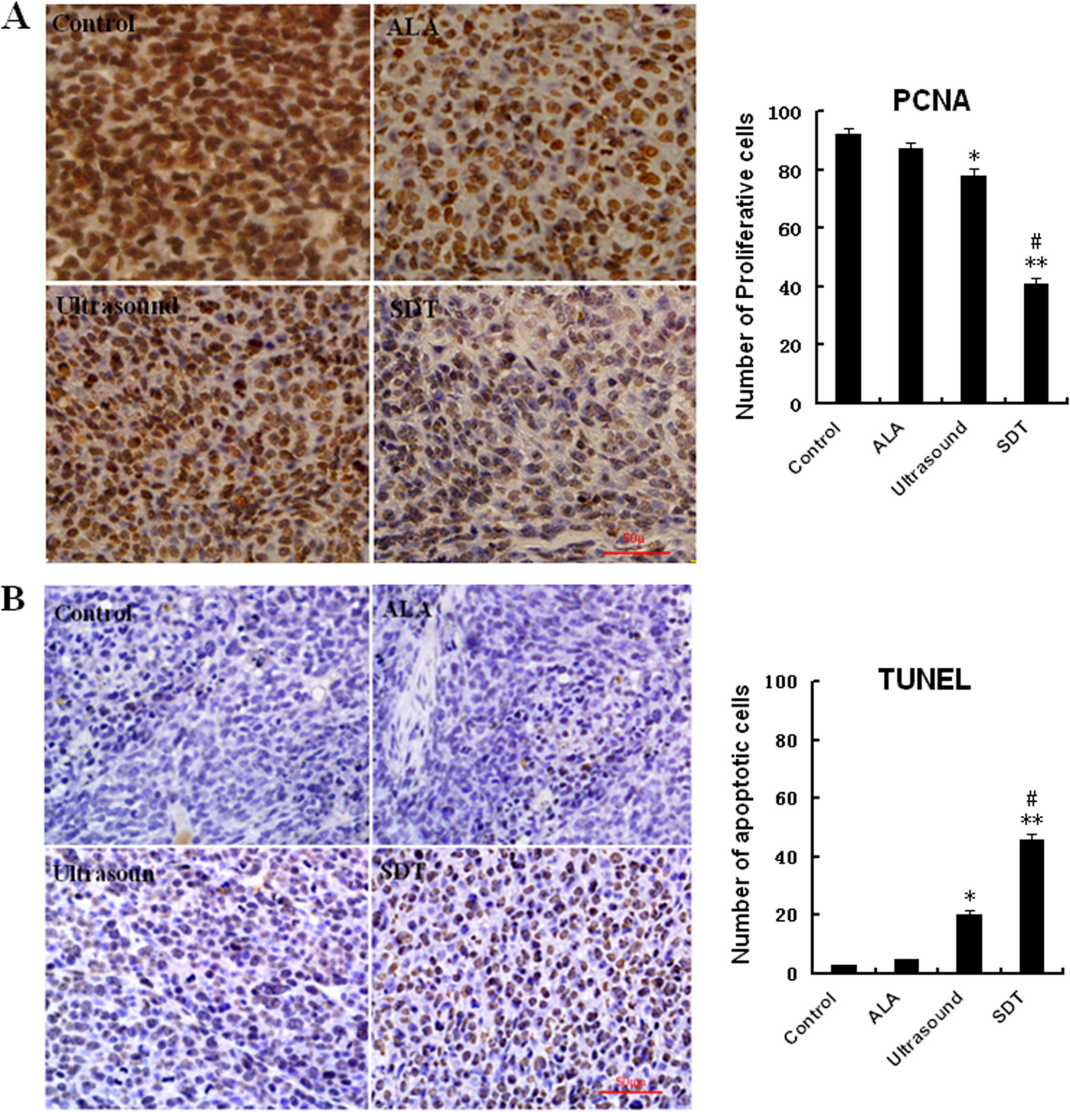
ALA-SDT inhibits the proliferation and promotes the apoptosis of implanted osteosarcoma cells in vivo. The PCNA expression and the frequency of apoptotic cells in tumor sections from the different groups of mice were characterized by immunohistochemistry and TUNEL assays, respectively. Data are representative images (magnification x 400) or expressed as the mean % ± SD of in individual groups of tumors (n = 10 per group). A. Immunohisochemsitry for PCNA expression. B. Quantitative analysis of the frequency of PCNA+ tumor cells. C> The TUNEL analysis of apoptotic cells in the tumors. D. The Quantitative analysis of apoptotic tumor cells. *P<0.05, **p<0.01 vs. the Control. #p<0.05 vs. the ultrasound alone.

To understand the therapeutic mechanisms of ALA-SDT in vivo, we analyzed the proliferative osteosarcoma cells in the implanted tumors by anti-PCNA staining. As shown in Fig 6A, in comparison with that in the Control group of tumors, ultrasound, but not 5-ALA, treatment significantly reduced the frequency of anti-PCNA positively stained proliferative osteosarcoma cells (p<0.05) and ALA-SDT further reduced the percentages of proliferative osteosarcoma cells (p<0.01). More importantly, the percentages of anti-PCNA positively stained proliferative osteosarcoma cells in the ALA-SDT group of tumors were significantly lower than that of the ultrasound-treated tumors (p<0.05). Hence, treatment with 5-ALA enhanced the inhibition of ultrasound on the proliferation of osteosarcoma cells in vivo.

Further analysis revealed that treatment ultrasound, but not 5-ALA alone, induced 20.4% of osteosarcoma cell apoptosis and ALA-SDT increased the percentages of apoptotic osteosarcoma cells to 45.6%, which was significantly higher than that in the Control (p<0.01) and the ultrasound alone (p<0.05). Together, our data indicated that the ALA-SDT inhibited the proliferation of implanted osteosarcoma cells and induced their apoptosis in vivo.

### The ALA-SDT modulates the expression of apoptosis-related regulators in the implanted osteosarcoma in vivo

**Fig 7:**
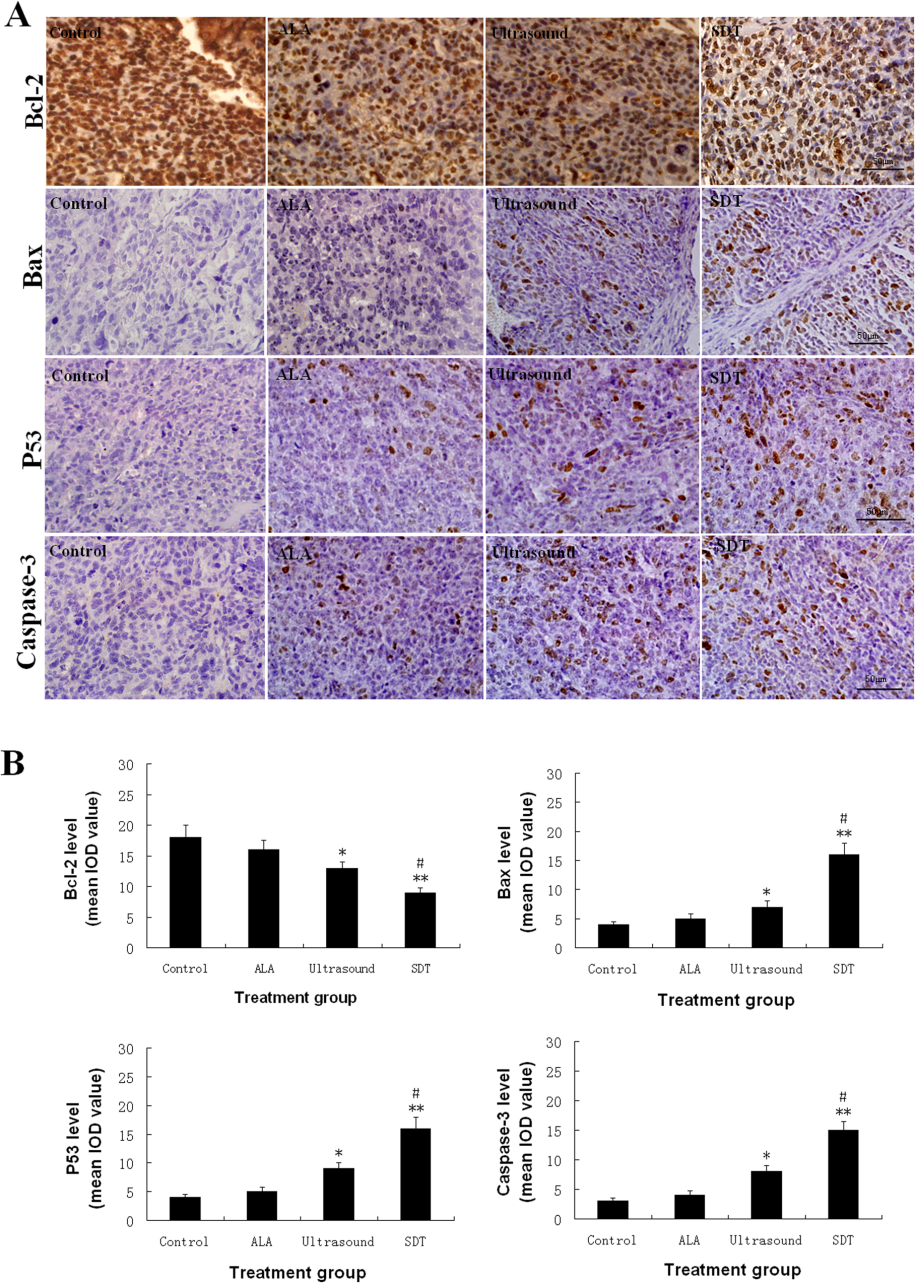
Immunohistochemsitry analysis of the levels of apoptosis-related regulators in the tumors. The levels of BcL-2, Bax, p53 and caspase 3 expression in the tumor sections from the different groups of mice were characterized by immunohistochemistry. Data are representative images (magnification x 200) or expressed as the mean ± SD of the IOD from the different groups of mice (n = 10 per group). A. Immunohisochemsitry analysis. B. Quantitative analysis of the IOD values. *P<0.05, **p<0.01 vs. the Control. #p<0.05 vs. the ultrasound alone.

The levels of p53, BcL family members and caspase expression are associated with the proliferation and apoptosis of tumor cells[15]. We further examined the expression levels of BcL-2, Bax, p53 and caspase 3 in the implanted tumors from the different groups of mice by immunohistochemistry (Fig 7A). Quantitative analysis of the intensity of anti-BcL-2 staining indicated that there was no significant difference in the intensity of BcL-2 expression between the Control and ALA groups of tumors (Fig 7B). The levels of BcL-2 expression in the tumors form the ultrasound groups were significantly lower than that in the Control (p<0.05), but significantly higher than that in the tumors from the ALA-SDT group of mice (p<0.05). In contrast, the levels of Bax, p53 and caspase 3 expression in the tumors from the ultrasound group were significantly higher than that in the Control (p<0.01), but significantly lower than that in the ALA-SDT group of mice (p<0.05). Collectively, these data indicated that treatment with 5-ALA enhanced ultrasound-mediated inhibition of BcL-2 expression, but increased ultrasound-up-regulated Bax, p53 and caspase 3 expression in the implanted osteosarcoma in mice.

## Discussion

In this study, we investigated the effects of ALA-SDT on the survival and apoptosis of rat osteosarcoma UMR-106 cells *in vivo* and *in vitro*. Firstly, we found that treatment with 5-ALA caused the maximum metabolic PpIX signals at 8 hours post treatment in the implanted osteosarcoma in mice, consistent with a previous report, suggesting the slow transition process from PpIX to heme[19]. Treatment with 5-ALA at <4 mM did not affect the survival of rat osteosarcoma UMR-106 cells and peaked the PpIX signals at 6 hours post treatment. Furthermore, treatment with 2 mM 5-ALA and low-intensity ultrasound for 7 minutes resulted in the maximum reduction in the viability of UMR-106 cells in vitro. The low-intensity ultrasound in our study was less than theoretical 3.0 W/cm^2^ standard and it should cause little thermal effect, rather result in the cavitation effect[20–24]. These conditions were used for subsequent experiments.

Previous studies have shown that different types of SDT can effectively inhibit the growth of tumors in humans and in animals[25,26]. In this study, we employed the optimized experimental conditions and we found that ALA-SDT significantly inhibited the growth of rat osteosarcoma in mice and reduced the survival rates of UMR-106 cells, as compared with that of controls and even with that of ultrasound alone. In addition, the ALA-SDT significantly reduced the percentages of PCNA+ rat osteosarcoma cells in the tumors. These data indicated that treatment with 5-ALA enhanced ultrasound-mediated inhibition of rat osteosarcoma cell proliferation in vivo and in vivo. More importantly, 5-ALA can be metabolized into a sonosensitizer. In comparison with other sonosensitizers, such as hematoporphyrin monomethyl ether (HMME) and thiocyanate B, 5-ALA has the advantages of fast metabolism, low toxicity and relatively high safety and 5-ALA has been used in the clinical practice. To the best of our knowledge, this was the first report that ALA-SDT inhibited the growth of implanted rat osteosarcoma in mice. Our data may provide an experimental basis for the design of new therapies for patients with osteosarcoma in the clinic.

SDT can induce ROS production and mitochondrial damages, and promote tumor cell apoptosis. Although ultrasound alone induced moderate frequency of tumor cell apoptosis, the ALA-SDT significantly enhanced the effect of ultrasound-triggered rat osteosarcoma cell apoptosis in vivo and in vitro, which were associated with the inhibition of osteosarcoma growth in vivo. Evidentially, we found that in comparison with that of ultrasound alone, ALA-SDT increased the percentages of UMR-106 cells and the severity of apoptosis-related cell structural damages and the numbers of apoptotic bodies in UMR-106 cells. Second, the ALA-SDT significantly reduced the ΔψM and increased the levels of ROS production in UMR-106 cells. Furthermore, the ALA-SDT significantly reduced the levels of BcL-2 expression, but elevated the levels of Bax, p53 and caspase 3 in the tumors. These support the notion that the ALA-SDT damages the mitochondria and through the mitochondrial pathway induces UMR-106 cell apoptosis[17,27]. It is well known that activation of sonosensitizers can induce ROS production in cells[8,27] and the high levels of ROS can damage the mitochondrial membranes and oxidize mitochondrial proteins, leading to depletion of antioxidants and formation of mitochondrial permeability transition pore as well as apoptosis [18,28]. In addition, high levels of ROS can convert into H_2_O_2_ and other toxic metabolites that release cytochrome C to activate caspase cascade, and finally lead to apoptosis[29,30]. Given that ultrasound alone moderately inhibited osteosarcoma cell proliferation and induced tumor cell apoptosis the mechanical effect of ultrasound may directly damage cell membranes and enhance cell membrane permeability[31,32]. This, together with the potential activation of endogenous PpIX to produce ROS by ultrasound, may trigger some tumor cell apoptosis[30]. In the presence of 5-ALA, the same intensity of ultrasound increased the levels of ROS production and the mechanical and cavitation effects, and enhanced antitumor activity of ultrasound by inducing tumor cell apoptosis through the mitochondrial pathway[4,16].

In summary, our data indicated that low intensity ultrasound alone significantly inhibited osteosarcoma cell proliferation and osteosarcoma growth in mice, associated with inducing osteosarcoma cell apoptosis. Treatment with 5-ALA enhanced ultrasound-mediated inhibition of osteosarcoma cell proliferation and ultrasound-induced osteosarcoma cell apoptosis in vivo and in vitro, associated with reducing the ΔψM and BcL-2 expression and increasing the levels of ROS production, Bax, p53 and caspase 3 expression. Our data suggest that the ALA-SDT through the mitochondrial pathway induces osteosarcoma cell apoptosis, leading to inhibition of implanted rat osteosarcoma growth in vivo. Our novel findings may provide experimental basis for the design of new therapies for intervention of osteosarcoma.
